# Relationship between dry eye disease and myopia: A systematic review and meta-analysis

**DOI:** 10.1016/j.heliyon.2024.e38674

**Published:** 2024-09-28

**Authors:** Xinrong Zou, Ken Nagino, Alan Yee, Akie Midorikawa-Inomata, Atsuko Eguchi, Shintaro Nakao, Hiroyuki Kobayashi, Takenori Inomata

**Affiliations:** a Juntendo University Graduate School of Medicine, Department of Ophthalmology, Tokyo, Japan; b Department of Ophthalmology, Fengcheng Hospital, Fengxian District, Shanghai, China; c Juntendo University Graduate School of Medicine, Department of Hospital Administration, Tokyo, Japan; d Juntendo University Graduate School of Medicine, Department of Digital Medicine, Tokyo, Japan; e Juntendo University Graduate School of Medicine, Department of Telemedicine and Mobile health, Tokyo, Japan; f Juntendo University Graduate School of Medicine, AI Incubation Farm, Tokyo, Japan

**Keywords:** Dry eye disease, Myopia, On-screen time, Younger population, Systemaric review, Meta-analysis

## Abstract

**Background/objectives:**

Dry eye disease (DED) and myopia are common ocular disorders. This systematic review and meta-analysis investigated the association between DED and myopia.

**Methods:**

PubMed and EMBASE were searched for articles published between 1984 and 2022. Study quality was assessed using the Joanna Briggs Institute Critical Appraisal Checklist, and analysis was conducted using the DerSimonian–Laird random-effects model.

**Results:**

Of the 1,313 studies identified, 15 studies on DED and myopia were included. The meta-analysis revealed that the overall prevalence of subjective DED symptoms in the myopia population was 45.1 % (95 % confidence interval: 0.287–0.616). There was a significant association between DED and myopia. The myopia population had higher Ocular Surface Disease Index scores and shorter tear film breakup times than the non-myopia population. Additionally, the meta-regression analysis showed that spherical equivalent was significantly associated with the prevalence of DED symptoms in adults with myopia.

**Conclusion:**

Interventions to prevent DED are required in the myopia population. Enhancing patient awareness and self-management for DED, in addition to early screening and detection, is especially critical for younger populations who are at a higher risk of developing myopia.

## Highlights

• Dry eye disease is significantly associated with myopia.
• Patients with myopia showed a 45.1 % prevalence of dry eye disease symptoms.
• Preventive interventions are important for dry eye disease in myopia population.
• Early screening and detection of dry eye disease are crucial for younger individuals at a higher risk of developing myopia.

## 1 Introduction

Dry eye disease (DED) is a common condition that negatively impacts quality of life and work productivity [1,2]. DED causes ocular discomfort, including dryness, foreign body sensation, eye fatigue, eye pain, and vision fluctuations, sometimes leading to bacterial infections, scarring, and corneal perforations [[2], [3], [4]]. DED is a multifactorial disorder of the ocular surface characterized by a loss of homeostasis of the tear film [5]. Interestingly, uncorrected refractive errors are a risk factor of DED [6].

Myopia is highly prevalent with 4.75 billion people estimated to be affected by 2050, which correlates to 49.8 % of the global population [7]. Myopia is prevalent in 80.0%–90.0 % of young adults in East Asia [8,9]. The prevalence of myopia is increasing considerably, and it has become an important public health issue [7,10].

DED and myopia frequently occur simultaneously [[10], [11], [12]]. Young children and adolescents have considerably increased screen time, shorter outdoor activity time, and increased time spent in near-view activities, all of which are risk factors for myopia [13,14]. Increased on-screen time and near-view work results in reduced blink rates and incomplete eyelid closure, thereby inducing and exacerbating DED symptoms [[15], [16], [17], [18], [19], [20]]. DED is a multifactorial disease that progresses with age [[21], [22], [23]]. Additionally, DED is associated with myopia [24,25]. Therefore, the assessment of DED in young patients with myopia deserves adequate attention.

DED and myopia have become common global healthcare issues [[26], [27], [28]]. Therefore, understanding their common pathogenesis and risk factors may lay the foundations for appropriate interventions for a significant population. This systematic review and meta-analysis aimed to summarize the current literature on the association between DED and myopia.

## 2 Materials and methods

### 2.1 Outcomes

Relationship between DED and myopia was systematically evaluated by reviewing published studies. Primary outcome of interest was the prevalence of DED among individuals with myopia.

### 2.2 Search strategy

This study followed the Preferred Reporting Items for Systematic Reviews and Meta-Analyses protocols [29]. Because all the reported data were obtained from the available published literature, neither institutional review board approval nor informed consents were required for this study.

**Fig. 1:**
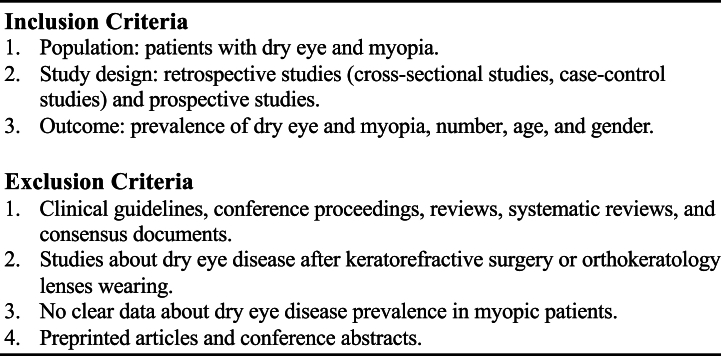
Inclusion and exclusion criteria for the systematic review.

A literature search was performed using free text terms and medical subject headings (MeSH in PubMed and Emtree in EMBASE) from PubMed and EMBASE articles published from January 1, 1976, to September 16, 2022. In the EMBASE search, gray literature was also included. Search formula was as follows: [((dry eye) AND (myopia)) OR ((dry eye) AND (refractive error))]. Fig. 1 lists the inclusion and exclusion criteria. All study designs, including retrospective, prospective, cross-sectional, case-control, and case series, were included in the review.

### 2.3 Data extraction

Data from eligible studies were extracted by two independent reviewers (X.Z. and K.N.) using a standardized data extraction sheet, and the results were cross-checked. The following data were collected: first author name, country, date of publication, study design, group size, and the characteristics of the participants, including age and sex, main results, and findings. Disagreements between the reviewers regarding the extracted data were resolved by a third reviewer (T.I.).

### 2.4 Study quality assessment

Study quality was assessed using the Joanna Briggs Institute (JBI) Critical Appraisal Checklists for Analytical Cross-Sectional Studies and Case Control studies [30]. The questionnaires consisted of questions answered with “yes”, “no”, “unclear”, or “not applicable”. The quality of the literature was determined based on the percentage of total number of “yes” responses, with ≥ 70.0 %, 69.0 %–50.0 %, and < 50.0 % indicating high, moderate, and low quality, respectively [31,32].

### 2.5 Statistical analyses

Prevalence of subjective symptoms of DED in patients with myopia was combined using a one-group meta-analysis [32,33] via the DerSimonian–Laird random-effects model [34]. Considering the large heterogeneity among the included studies, prevalence of clinical signs of DED in the myopia population was not investigated. The prevalence was estimated in adults (age ≥ 18 years), as well as teenagers and children (age < 18 years). Meta-regression analysis was performed to assess the association of age and spherical equivalent with the estimated prevalence of subjective DED symptoms.

Heterogeneity was assessed using I^2^ and the chi-square test. An I^2^ > 75.0 % was considered to indicate high heterogeneity, and *P* < 0.05 indicated significant heterogeneity [35]. When high heterogeneity was observed, a sensitivity analysis was performed by excluding studies suspected to be the cause of heterogeneity and those with low quality. Egger's and Begg's tests were performed to estimate publication bias, with *P* < 0.05 in both tests indicating significant publication bias [36,37]. The STATA software package (v. 17.0; Stata Corp, College Station, TX, USA) with the metaprop command was used for all analyses [38].

## 3 Results

### 3.1 Search results

**Fig. 2:**
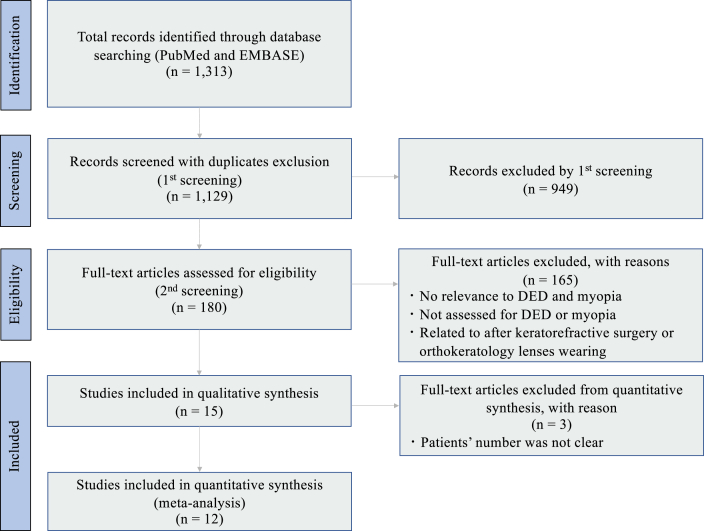
PRISMA flow diagram illustrating the selection process of articles.

Overall, 1,313 original research articles published between 1984 and 2022 were identified. After removing 184 duplicates, 1,129 articles were retained. Due to article type and subject irrelevance, 949 articles were excluded. Subsequently, 165 articles were excluded for wrong target population (n = 73), for not assessing DED prevalence in the myopia population (n = 30), or for being related to DED after keratorefractive surgery or orthokeratology lens use (n = 62). Finally, 15 articles were included in the systematic review (Fig. 2) [24,25,[39], [40], [41], [42], [43], [44], [45], [46], [47], [48]], and 12 in the meta-analysis. The studies by Fahmy et al., Yotsukura et al., and Upaphong et al. were excluded from the meta-analysis as the number of patients with DED in the myopia population was not reported [[49], [50], [51]].

### 3.2 Study and demographic characteristics

Fifteen papers [24,25,[39], [40], [41], [42], [43], [44], [45], [46], [47], [48], [49], [50], [51]] that examined DED in individuals with myopia were published between November 2003 and September 2022. Three studies were published in China [44,46,47], three in Saudi Arabia [45,48,49], three in Australia [[39], [40], [41]], two in Japan [25,50], and one each in Iran [42], Turkey [24], and Thailand [51], and Russia [43]. There were nine prospective non-interventional cross-sectional studies [25,[42], [43], [44],^46^,^47^,[49], [50], [51]], three case-control studies [24,45,48], and three retrospective case-control studies [[39], [40], [41]]. The subject size ranged from 25 to 1,849 patients, with a total of 7,336 patients. The age ranged from 4 to 73 years. Twelve studies reported the number of men (total = 2,433) and women (total = 3,235) [24,25,[39], [40], [41], [42], [43], [44], [45],^47^,^50^,^51^]. Of these 15 papers, eight studies included candidates for corneal laser refractive surgery for myopia [[39], [40], [41], [42], [43],^46^,^47^,^51^], four investigated the relationship between DED and myopia [24,45,48,49], two investigated the prevalence of DED in teenagers with myopia [44,50], and one investigated the association between DED and myopia in children [25]. Basic information (authors, publication date, and study design), sample size, patient demographic data, DED test results, prevalence of DED in adults and adolescents with myopia, results of study quality assessment, relationship between DED and myopia, and the remarks of each study are summarized in Table 1. Details of the study quality assessment results are presented in Supplementary Table 1.

**Table 1:** Study characteristics and main results.

Source	Publication date	Country	Study design	Group and sample size	Characteristics	Patients with DED	Outcomes in common	Quality score	Main results	Remarks
1. Ilhan et al. [24]	Feb. 2014	TR	Case-control study	45 myopia patients 44 controls	22 M (48.9 %) 23 F (51.1 %) 40.2 ± 12.3 y (18–63 y) 21 M (47.7 %) 23 F (52.3 %) 38.8 ± 9.3 y (22–57 y)	97.8 % (44/45) patients had DED symptoms with OSDI ≥ 13. 11 controls (25 %) had mild DED symptoms.	A correlation existed between pathologic myopia and DED.	90 % (high)	There was a significant difference between the groups in SE, keratometry, AL, TFBUT, and OSDI scores (*P* < 0.001). Group 1: SE 29.6 ± 3.8 D, keratometry 43.9 ± 1.1 D, and AL 27.4 ± 0.6 mm, TFBUT 7.2 ± 3.4 s, SIT and STA 14.4 ± 6.1 and 9.5 ± 4.5 mm, OSDI 60 % of the patients had severe OSDI symptoms. Group 2: SE 20.1 ± 0.5 D, keratometry 42.3 ± 1.4 D, and AL 23.0 ± 0.2 mm, TFBUT 13.6 ± 3.7s, SIT and STA 16.7 ± 6.2 and 11.4 ± 6.0 mm, OSDI 75 % of the patients had no ocular symptoms.	Patients with pathologic myopia had lower TFBUT scores and higher OSDI scores when compared with healthy individuals.
2. Hazra, et al. [25]	Jun. 2022	JP	Cross-sectional study	78 Children with mypopia	28 F (38.9 %) 44 M (61.1 %) 12.8 y ± 2.7 y (4–16 y)	94.9 % (74/78) children with myopia and DED symptoms	There was an association between DED and myopia in children with myopia and DED symptoms.	87.5 % (high)	The TFBUT was correlated significantly with the corneal HOAs and intraocular HOAs. Multiple regression analyses showed that the AL was associated significantly with the TFBUT (β = −0.067, *P* = 0.004), the intraocular HOAs, and total ocular HOAs. The CT was associated significantly with the TFBUT and AL (β = 9.15 and −7.85, respectively; *P* < 0.001 and = 0.01, respectively)	The association between DED and myopia might be independent of the HOAs, and the TFBUT was associated with the CT, which is related to the AL.
3. Albietz [39],	Nov. 2003	AU	Retrospective case control study	300 myopia patients	170 F (56.7 %) 130 M (43.3 %) 40.8 y ± 11.0 y	18.7 % (56/300) myopia patients diagnosed with DED before surgery.	Preoperative DED existed in the myopia population.	80.0 % (high)	18.7 % (56/300) of refractive candidates had DED before surgery. Significant inverse correlations existed between goblet cell density and DED symptoms.	Ocular surface management reduced DED symptoms after refractive surgeries.
4. Albietz et al. [40]	Mar. 2004	AU	Retrospective case control study	565 myopia patients	316 F (56.0 %) 249 M (44.0 %) 36.0 y ± 10.0 y (18–72 y)	25.1 % (142/565) patients reported DED symptoms before surgery.	Preoperative DED existed in the myopia population.	100 % (high)	18 % patients (102/565) reported DED symptoms; 7 % (40/565) were diagnosed with DED before surgery. Regression after LASIK occurred in 12 (27 %) of 45 patients with chronic dry eye and in 34 (7 %) of 520 patients without (*P* < 0.001).	Regression after LASIK was related to chronic DED.
5. Albietz et al. [41]	Mar. 2005	AU	Retrospective case control study	932 myopia patients	522 F (56.0 %) 410 M (44.0 %) 36.0y ± 9.0 y (18–65 y)	21.2 % (198/932) patients had DED symptoms before surgery.	Preoperative DED was common in myopia population.	100 % (high)	21.2 % (198/932) of myopia patients were diagnosed with DED before surgery.	The risk of chronic DED after LASIK was significantly higher in Asian eyes.
6. Farahi et al. [42]	Mar. 2014	IR	Prospective cross-sectional study	332 myopia patients	216 F (65 %) 116 M (35 %) 27.3 ± 6.1 y (18–50 y)	15.9 % (50/315) had DED symptoms with MQ > 10.	Some refractive surgery candidates had abnormal tear function, especially women and older individuals.	100 % (high)	The FBUT and STA were abnormal in 30.8 % and 14.6 % of cases. 15.9 % of patients showed abnormal MQ scores. MQ scores were significantly higher (*P* < 0.001) and FBUT was significantly lower in women (*P* = 0.003) and older ages in abnormal cases.	Some cases seeking keratorefractive surgery for myopia had abnormal tear function, especially women and older individuals.
7. Wang et al. [44]	Jan. 2016	CN	Prospective cross-sectional study	248 myopia patients	132 F (53.2 %) 116 M (46.8 %) 12.3 ± 1.9y (7–18y)	19.0 % (47/248) had DED symptoms with OSDI ≥ 13.	Teenagers with myopia also suffered from DED.	100 % (high)	The prevalence of DED in teenagers with myopia was 19.0 %. All three meibomian gland dysfunction parameters (i.e., meibomian gland orifice scores, meibomian gland secretion scores, and meibomian gland dropout scores) of the DED group were significantly higher than those of the normal group (*P* < 0.001).	Meibomian gland dysfunction plays an important role in DED in teenagers with myopia.
8. Maychuk [43].	Mar. 2016	RU	Prospective noninterventional cross-sectional study	400 myopia patients	263 F (65.8 %) 137 M (34.2 %) 29.7 ± 10y (18–73y)	62.2 % (248/399) had DED symptoms with OSDI ≥ 13.	Preoperative DED was common in the myopia population.	100 % (high)	36.5 % had evidence of tear-volume deficiency (Schirmer score ≤ 10 mm). 10.1 % of patients had tear-film instability (TFBUT < 5 s). The mean OSDI score was 20.4. DED severity (DEWS grading): mild/episodic 66.2 %, moderate 29.5 %, and severe 4.3 %.	DED prevalence in candidates for myopia surgery was estimated at approximately 10 %–40 % (based on clinical signs) and 40 %–55 % (based on symptoms).
9. Li et al. [46]	Jun. 2021	CN	Prospective noninterventional cross-sectional study	1,849 myopia patients	All sexes (No data for sex) 18–45y	57.1 % (1,055/1,849) had DED symptoms.	A high proportion of refractive surgery candidates have preexisting DED.	75 % (high)	41.4 % were diagnosed with DED (766/1,849). The overall mean TFBUT and SIT values were 7.3 ± 3.7 s and 15.2 ± 8.8 mm. The total prevalence of ocular surface fluorescein staining was 23.5 % (422/1,849). 44.6 % of patients had TFBUT < 5 s and 23.2 % of patients had SIT < 5 mm.	A high proportion of refractive surgery candidates have preexisting DED.
10. Alanazi et al. [45]	Jan. 2021	SA	Case control study	50 myopia patients	50 F 20.3 ± 1.1y (18–30y) 50 controls 22.2 ± 1.5y (18–30y)	48 % (24/50) had DED symptoms with OSDI ≥ 13.	Myopia in young females has a negative effect on tear film in terms of DED symptoms, tear volume.	80 % (high)	The myopia group had higher median scores of OSDI (13.5 [15.3] vs. 6.0 [4.0], *P* < 0.001), and TF (1.6 [1.3] vs. 0.9 [0.8], *P* < 0.001) compared to the control group. The median phenol red thread score (27.5 [6.3] vs. 29.5 [5.0], *P* = 0.003) was significantly lower in the myopia group than in the control group.	Myopia in young females has a negative effect on tear film in terms of DED symptoms, tear volume, and TF grades.
11. Zhao et al. [47]	Jul. 2021	CN	Prospective cross-sectional study	141 myopia patients	45 M (31.9 %) 96 F (68.1 %) 27.8 ± 6.1 y (18–45y)	45.4 % (64/141) patients had DED symptoms.	Preoperative DED was common in the myopia population.	100 % (high)	64 patients (45.4 %) were diagnosed with DED. The diopter level of DED patients was significantly higher (Z = −2.086, *P* = 0.019). For every 1D increase in diopter, the risk of DED increased by 0.761 times.	Preoperative DED is relatively common.
12. Fagehi et al. [48]	Feb. 2022	SA	Case-control study	25 Myopia patients	All sexes (No data for sex) 28.8y ± 6.8 y (18–38y)	36 % (9/25) myopia patients had DED with OSDI≥13.	Myopia patients had higher OSDI and tear evaporation rate scores than normal controls.	80 % (high)	The OSDI score and tear evaporation rate measurements showed DED symptoms in 36 % and 48 % of myopia subjects, respectively. Significant strong positive correlations were detected between the OSDI and tear evaporation rate scores in myopia (*P* = 0.004; *r* = 0.559) OSDI: myopia 11.0 (7.5) vs. control 5.0 (6.0), *P* < 0.001; tear evaporation rate scores: myopia 27.0 (22.7) vs. control 12.7 (7.8), *P* < 0.001.	The tear evaporation rate scores were significantly higher in subjects with myopia as compared with normal controls.
13. Fahmy et al. [49]	May 2018	SA	Prospective cross-sectional study	126 Participants	19–25 y (No data for sex) 49 emmetropic eyes (± 0.50 SE) 48 myopia eyes (≤ −0.75 SE) 31 hyperop-ic eyes (> +0.75 SE)	36.5 % had myopia 24.6 % had emmetropia 17.4 % had hypermetr-pia	Correlation existed between refractive errors and dryness level.	75 % (high)	The prevalence of DED was 24.6 %, 36.5 %, and 17.4 % in emmetropia, myopia and hypermetropia, respectively. NIBUT had a positive correlation with myopia, and the average NIBUT has a significant reduction in myopia.	They demonstrated a correlation between refractive errors and dryness level.
14.Yotsukura et al. [50]	Nov. 2019	JP	Cross-sectional study	1416 subjects (1,227 children with myopia)	792 F (55.9 %) 624 M (44.1 %) 10.8 y ± 2.7 y (6–14 y)	11.7 % (165/1,416) subjects with DED.	Their data suggested the possibility of an association between DED and myopia.	87.5 % (high)	The prevalence of DED in children with myopia was 11.7 %. The greater the symptoms of DED, the higher the myopia refraction was in the elementary school students and the longer the axial length was in the junior high school students.	There could be an association between DED and myopia.
15. Upaphong et al. [51]	Jan. 2022	TH	Cross-sectional study	1,167 myopia subjects	643 F (55.1 %) 524 M (44.9 %) 33.3 y ± 12.6 y (26–39 y)	208/2,334 eyes (8.91 %) with DED.	DED is one of the most common ocular comorbidities in refractive candidates.	87.5 % (high)	DED prevalence from self-reported symptoms 116/2,334 eyes (4.97 %); 208/2334 eyes (8.91 %) from screening by signs and/or symptoms. DED diagnosed by 2006 Japanese DED criteria.	Some refractive candidates were diagnosed with DED based on clinical signs rather than symptoms.

Abbreviations: AL, axial length; AU, Australia CN, China; CT, choroidal thickness; DED, dry eye disease; F, female; FBUT, fluorescein breakup time; HOAs, higher-order aberrations; IR, Iran; JP, Japan; M, male; MQ, McMonnies DED questionnaire; NIBUT, non-invasive tear breakup time; OSDI, Ocular Surface Disease Index; RU, Russia; SA, Saudi Arabia; SE, spherical equivalence; SIT, Schirmer's test without anesthesia; STA, Schirmer's test with anesthesia; TFBUT, tear film breakup time; TF, tear ferning; TH, Thailand; TR, Turkey.

### 3.3 Prevalence of diagnosed DED and myopia in adults

Prevalence of DED in refractive surgery candidates varied from 18.7 % (56/300) to 45.4 % (64/141) [39,47]. In a cross-sectional study in Russia [43], the prevalence of DED was approximately 10.0–40.0 % in the preoperative myopia population (based on clinical signs). In a multicenter study in China [46], DED was diagnosed in 41.4 % (766/1,849) of refractive surgery candidates. Farahi et al. [42] reported that 30.8 % (100/325) of cases had abnormal tear film breakup time (TFBUT), and 14.6 % (47/325) had abnormal tear secretion volume, as determined by Schirmer's tests. In a study in Thailand, the prevalence of DED was 8.9 % (208/2,334 eyes) in refractive surgery candidates. However, this study calculated the prevalence per eye, and accurately measuring the prevalence of DED in myopia populations was challenging [51].

Apart from DED prevalence in refractive surgery candidates, prevalence of DED was also relatively higher in the ordinary myopia population than in the control group, including in subjects with emmetropia and hypermetropia [45,49]. Fagehi et al. [48] found that subjects with myopia had significantly higher tear evaporation rates than controls, and there was a significant positive correlation between the Ocular Surface Disease Index (OSDI) score and the tear evaporation rate score in patients with myopia (*P* = 0.004, *r* = 0.559). Fahmy et al. [49] found that DED was prevalent in 24.6 % (31/126), 36.5 % (46/126), and 17.4 % (22/126) of patients with emmetropia, myopia, and hypermetropia, respectively. Alanazi et al. [45] divided their patients (n = 50) into two groups based on myopia severity: mild (−0.25 to −3.00 diopter) and moderate (−3.10 to −6.00 diopter). Among the 50 patients, 48.0 % experienced symptoms of DED according to OSDI scores, and 36.0 % had DED according to tear ferning (TF) grades. These two groups had significantly higher median OSDI and TF scores than the control group. However, severity of DED was not correlated with the myopia severity [45].

Tear film instability is widespread in patients with DED and myopia [24,47,49] Zhao et al. [47] found that the odds ratio (OR) of DED increased by 0.76 [OR: 1.76 (95 % CI: 1.55–1.98)] for every 1 diopter increase. Ilhan et al. [24] reported that patients with pathologic myopia had lower TFBUT and higher OSDI scores than healthy participants, speculating a relationship between DED and pathologic myopia. Furthermore, Fahmy et al. [49] reported that non-invasive TFBUT was positively correlated with myopia. Thus, myopia is closely associated with the occurrence of DED.

### 3.4 Prevalence of DED in teenagers and children with myopia

Only three articles discussed pediatric myopia, likely owing to poor cooperation with DED examinations. Wang et al. [44] reported a DED prevalence of 19.0 % (47/248) in myopia teenagers based on OSDI scores ≥ 13 [52]. Two Japanese studies revealed a relationship between DED and myopia in teenagers and children. Yotsukura et al. [50] investigated the prevalence rate of schoolchildren with myopia and found that the severity of DED symptoms was positively correlated with the degree of myopia refraction in elementary school students and with longer axial lengths in junior high school students. Hazra et al. [25] analyzed the association between DED and myopia in 78 children with myopia with DED symptoms and revealed that TFBUT was associated with choroidal thickness, which was related to axial length. These results indicate an association between DED and myopia.

### 3.5 Comparison of DED parameters in myopia and control groups in three case-control studies

Ilhan et al. [24] found a difference in TFBUT between the myopia and control groups (7.2 ± 3.4 s vs. 13.6 ± 3.7 s, *P* < 0.001). Among patients with myopia, 97.8 % (44/45) had an OSDI score ≥ 13, compared to 25.0 % (11/44) of controls (*P* < 0.001). Alanazi et al. [45] discovered that the myopia group had a higher median OSDI score (13.5 points, [interquartile range, 15.3], vs. 6.0 [4.0] points, *P* < 0.001) and TF grade (1.6 [1.3] points vs. 0.9 [0.8] points, *P* < 0.001) than control. In addition, the median phenol red thread scores were lower in the myopia group than the control group (27.5 [6.3] mm vs. 29.5 [5.0] mm, *P* = 0.003). Fagehi et al. [48] found that the myopia group had higher OSDI scores (11.0 [7.5] points vs. 5.0 [6.0] points, *P* < 0.001) and tear evaporation rate scores (27.0 [22.7] mm vs. 12.7 [7.8] mm, *P* < 0.001) than the control group. In summary, these findings suggest an association between myopia and increased risk of DED symptoms and ocular surface disorders.

### 3.6 Meta-analysis of prevalence of subjective DED symptoms in the myopia population

**Fig. 3:**
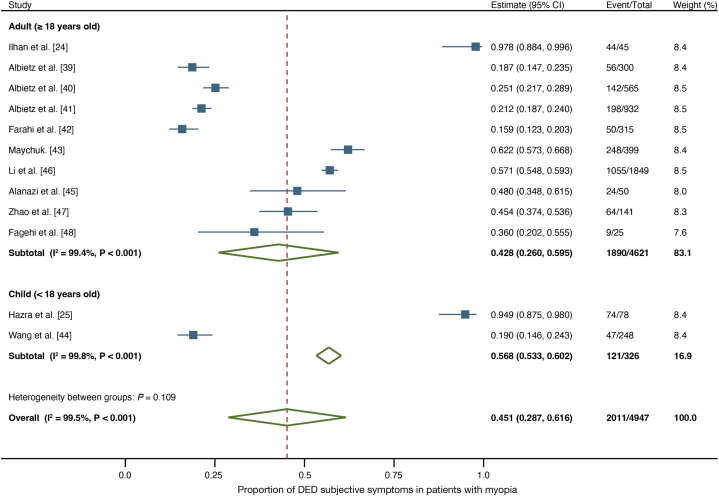
Forest plot of DED prevalence based on subjective symptoms in adults and children with myopia. The estimated prevalence of subjective symptoms of DED is 42.8 % for adults, 56.8 % for children, and 45.1 % overall in the myopic population.

Fig. 3 shows the forest plots illustrating a one-group meta-analysis of the 12 studies. Overall prevalence of subjective symptoms of DED within the myopia population was 45.1 % (2,011/4,947; 95 % confidence interval [CI]: 0.287–0.616). In adults, the prevalence of subjective DED symptoms within the myopia population was 42.8 % (1,890/4,621; 95 % CI: 0.260–0.595), while that of the pediatric group was 56.8 % (121/326; 95 % CI: 0.533–0.602). Publication bias was not observed (Begg's test: *P* = 0.451; Egger's test: *P* = 0.940).

**Fig. 4:**
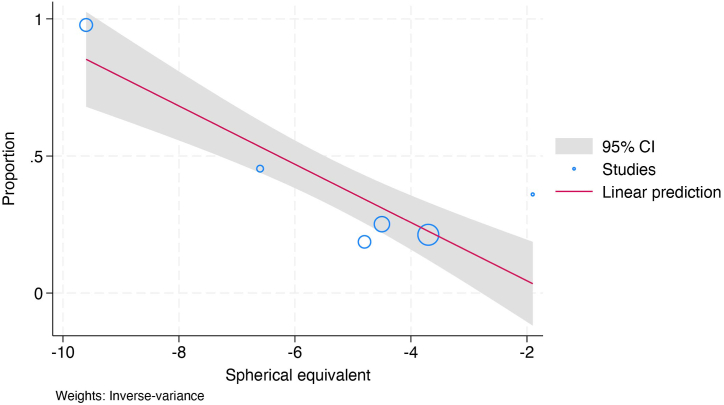
Bubble plot with a fitted meta-regression line of the correlation between the spherical equivalent and the prevalence of the subjective symptoms of DED. The plot shows that a tendency for a smaller spherical equivalent, indicating more severe myopia, is associated with a higher prevalence of subjective DED symptoms.

In the meta-regression analysis on the association between spherical equivalent and prevalence of subjective symptoms of DED, the pediatric group was excluded due to insufficient number of studies [25,50]. A negative correlation was observed between the spherical equivalent and prevalence of DED symptoms in adults (coefficient, −0.106, 95 % CI: −0.142 to −0.070) (Fig. 4).

**Fig. 5:**
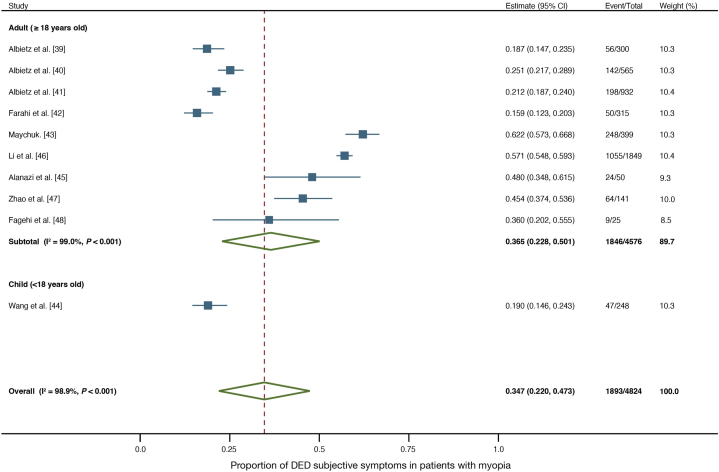
Forest plot of the prevalence of subjective symptoms of DED with sensitivity analysis. The estimated prevalence of subjective symptoms of DED is 36.5 % for adults, 19.0 % for children, and 34.7 % overall in the myopic population, after excluding studies with outliers.

**Fig. 6:**
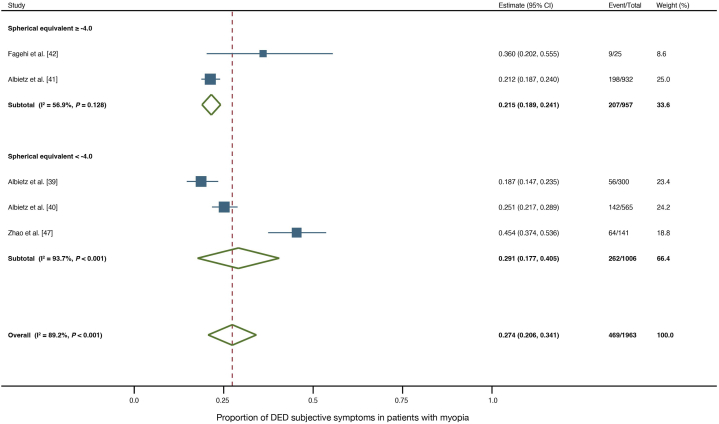
Forest plot of the prevalence of subjective symptoms of DED by subgroup analysis of spherical equivalent. Among the study participants who reported having a spherical equivalent, the estimated prevalence of subjective DED symptoms is 21.5 % for the group with a spherical equivalent ≥ −4.0, 29.1 % for the group with a spherical equivalent < −4.0, and 27.4 % overall.

Sensitivity analyses were performed to address the high heterogeneity. Two studies with prevalence of DED symptoms exceeding 90.0 % were excluded for concerns of being outliers leading to high heterogeneity [24,25]. In addition, a subgroup analysis was performed on studies with a reported spherical equivalent of ≥ −4.0 and those with < −4.0, based on the spherical equivalent showing an association with the prevalence of DED symptoms in the meta-regression. Prevalence of DED symptoms was 34.7 % after excluding the studies suspected of causing heterogeneity (Fig. 5). Subgroup analysis based on spherical equivalent showed that high heterogeneity was resolved in the subgroup with spherical equivalent ≥−4.0 (Fig. 6).

## 4 Discussion

This systematic review and meta-analysis found that the prevalence of subjective DED symptoms was 45.1 % (95 % CI: 0.287–0.616) in the myopia population. The myopia population had lower TFBUT and higher OSDI scores than the non-myopia population. In addition, the spherical equivalent was associated with the prevalence of DED symptoms in the adult myopia group. These results may indicate an association between DED and myopia, warranting close attention to signs of DED in patients with myopia since early screening and detection can help prevent the onset or progression.

This systematic review showed that DED and myopia share some common features. First, both are highly prevalent, particularly in Asia [7,9,53]. Over the past 60 years, the incidence of myopia among school-aged children in East and Southeast Asia has markedly increased [54]. Currently, up to 90 % of young adults in mainland China have myopia [9]. The “boom of myopia [10]” can also be seen in other East and Southeast Asian countries, including Japan, Singapore, and South Korea [12,[54], [55], [56]]. In another study, prevalence of symptomatic DED with or without clinical signs was 5%–50 % [11]. The high prevalence of DED and myopia has led to significant overlap between the two patient populations.

Second, this systematic review identified shared risk factors for DED and myopia, such as prolonged screen use and close-up work [57,58]. Increased digital screen time is associated with abnormal blinking and evaporative DED [[59], [60], [61], [62]]. Each additional hour of daily digital screen exposure correlates with 15.0 % increased odds of DED [18]. Extended screen time indirectly influences myopia risk by impacting sedentary behaviors, with a 10.0 % increase in screen time associated with a 2.9 % increase in myopia incidence among college students [60]. Prolonged device use can alter the morphology of the meibomian glands in myopia children, a factor closely associated with DED [19]. Consequently, it is crucial to recognize the shared risk of prolonged screen use for both DED and myopia, emphasizing preventive measures such as frequent breaks, blinking habits, and outdoor activities [56,61,62].

Near-view activities are correlated with myopia [58,[63], [64], [65]]. Each additional diopter-hour of weekly near-view work increases myopia risk by 2.0 % (OR: 1.02, 95 % CI: 1.01–1.03) [63]. Myopia progression is associated with near-view work within 30 cm (*P* = 0.001) and continuous near-view work exceeding 30 min (*P* = 0.023) [64]. Prolonged near-view work is linked to a 92.0 % myopia prevalence and DED in university students [66]. DED is highly prevalent in manufacturing workers with reduced blink rates during near-view tasks (OR: 4.22, 95 % CI: 1.62–10.95) [67]. These findings underscore the increased risk of myopia and DED with prolonged near-view activities, emphasizing the need for precautions such as avoiding extended eye use, maintaining proper posture, and increasing outdoor time [56,59].

Using meta-regression, we performed a subgroup analysis on studies reporting a spherical equivalent of ≥ −4.0 and < −4.0 and found an association between spherical equivalent and prevalence of DED symptoms in myopia adults. This association is further highlighted by the characteristics of high myopia, marked by an elongated ocular axis and increased intraocular volume, frequently causing exophthalmos and proptosis [68,69]. These alterations affect tear dynamics [70,71], causing tear evaporation and eye dryness [72,73]. Tsubota et al. demonstrated that tear evaporation is proportional to the exposed ocular surface area, in association with increased fissure width and lipid layer thinning [74]. Thinner aqueous and mucin layers contribute to lower TFBUT due to higher surface tension. Proptosis exacerbates DED symptoms and the risk of exposure keratopathy [75]. Consequently, patients classified as being highly myopic may have an increased risk for DED with a reasonable mechanistic explanation between their connection.

DED has been linked to myopia through higher-order aberrations in the parasympathetic nervous system, suggesting common developmental factors [25]. Molecularly, both myopia and DED are associated with miR-328 overexpression [76]. miR-328 may influence myopia development by mediating the *PAX6* gene [76]. Anti-miR-328 has been shown to protect corneal cells and accelerate re-epithelialization [77], indicating a potential relationship between DED and myopia.

Consistent findings, including those from three comprehensive case-control studies [24,45,48], also highlight a significant link between DED and myopia. With proper recognition of their comorbidity and timely interventions, clinicians can help promote ocular health for patients with DED and myopia.

As the prevalence of DED and myopia is expected to increase, managing co-risk factors is crucial in breaking the cycle between the two conditions. Interventions for the pediatric myopia population should involve strengthened collaborations between parents and schools. Early recognition, awareness, and self-management of DED are critical for patients with myopia, and clinician-led early screening and detection may play a pivotal role.

This study had several limitations. First, variations in diagnostic criteria and testing procedures for detecting DED among included studies introduce inconsistencies. Such methodological differences may lead to disparities in objective clinical indicators. Second, the analysis did not differentiate patients wearing soft contact lenses due to ambiguous descriptions in the 15 included studies. This may potentially impact the relationship between DED and myopia as contact lens use can induce or accelerate DED [78]. Third, there was significant heterogeneity among the included studies, potentially stemming from differences in patient characteristics and geographic locations. Thus, the findings should be interpreted cautiously. Particularly, meta-regression and sensitivity analyses showed an association between the spherical equivalent and prevalence of DED symptoms among adults with myopia; however, studies that did not report spherical equivalents may have contributed to the high heterogeneity. Lastly, owing to the limited number of studies evaluating DED in patients with myopia, we did not restrict the study designs included in the analysis. This lack of restriction may have increased heterogeneity owing to the inclusion of diverse study designs. To address potential heterogeneity, a random-effects model was used, allowing for the analysis of studies with different designs. In addition, sensitivity analyses were performed by excluding studies contributing to heterogeneity, and subgroup analyses were conducted under different conditions. Consequently, a more comprehensive body of evidence may have been provided.

In conclusion, DED is associated with myopia, and the prevalence of subjective DED symptoms was 45.1 % (95 % CI: 0.287–0.616) in the myopia population. Enhancing awareness of their comorbidity, promoting self-management of DED in patients with myopia, and prompt screening and detection of DED by clinicians may be effective in reducing the prevalence of DED in individuals with myopia. Timely interventions for DED and myopia can also help reduce overall economic burden for their management.

## Data availability statement

The data generated or analyzed in this study are included in this article.

## Funding

This research was supported in part by 10.13039/501100001691JSPS KAKENHI Grant Numbers 20KK0207 (TI), 22K16983 (AE), 23K16364 (AMI), 23K18406 (TI), and 24K19796 (AE).

## CRediT authorship contribution statement

**Xinrong Zou:** Writing – review & editing, Writing – original draft, Investigation, Formal analysis, Data curation. **Ken Nagino:** Writing – review & editing, Writing – original draft, Investigation, Formal analysis, Data curation. **Alan Yee:** Writing – review & editing, Writing – original draft. **Akie Midorikawa-Inomata:** Writing – review & editing, Writing – original draft, Methodology, Funding acquisition. **Atsuko Eguchi:** Writing – review & editing, Writing – original draft, Validation, Funding acquisition. **Shintaro Nakao:** Writing – review & editing, Writing – original draft, Validation. **Hiroyuki Kobayashi:** Writing – review & editing, Writing – original draft, Validation. **Takenori Inomata:** Writing – review & editing, Writing – original draft, Validation, Methodology, Investigation, Funding acquisition, Conceptualization.

## Declaration of competing interest

The authors declare the following financial interests/personal relationships which may be considered as potential competing interests: K.N. and A.M.I. received personal fees from InnoJin, Inc., outside the submitted work. S.N. received consulting fee from Chugai Pharmaceutical Co., Ltd., Kowa Company, Ltd., Novartis Pharma KK., Riverfield Inc., travel reimbursements and speaker fees from Alcon Inc., Boehringer Ingelheim Co., Ltd., Bayer Yakuhin Ltd., Canon Inc., JFC Sales Plan Co., Ltd., Kowa Company, Ltd., Mitsubishi Tanabe Pharma Corporation, Machida Holdings Inc., MSD K.K., Novartis Pharma KK., Novo Nordisk Pharma Ltd., Otsuka Pharmaceutical Co., Ltd., Santen Pharmaceutical Co., Ltd., Senju Pharmaceutical Co., Ltd., and Wakamoto Pharmaceutical Co., Ltd. not related to the submitted work. T.I. received non-financial support from Lion Corporation and Sony Network Communications Inc.; grants from Johnson & Johnson Vision Care Inc., Yuimedi Inc., ROHTO Pharmaceutical Co. Ltd., Kobayashi Pharmaceutical Co. Ltd., Kandenko Co. Ltd., and Fukoku Co. Ltd.; and personal fees from Santen Pharmaceutical Co. Ltd., InnoJin Inc., and Ono Pharmaceutical Co., Ltd. not related to the submitted work. The remaining authors declare no competing interests. The sponsors had no role in the design or execution of the study; data collection and management; the analysis and interpretation of the data; the preparation, review, or approval of the manuscript; or the decision to submit the manuscript for publication. If there are other authors, they declare that they have no known competing financial interests or personal relationships that could have appeared to influence the work reported in this paper.
