# Supplementary material for: Relationship between dry eye disease and myopia: A systematic review and meta-analysis
Source: Heliyon. 2024 Sep 28;10(19):e38674. doi: 10.1016/j.heliyon.2024.e38674 (PMC11471511; doi:10.1016/j.heliyon.2024.e38674)
Supplement: Multimedia component 1 [file mmc1.pdf]

**Relationship between Dry Eye Disease and Myopia: A Systematic Review and Meta-analysis**

Xinrong Zou MD; Ken Nagino PhD; Alan Yee, PhD; Akie Midorikawa-Inomata, RN, MPH, PhD; Atsuko Eguchi, PhD; Shintaro Nakao, MD, PhD, Hiroyuki Kobayashi, MD, PhD; Takenori Inomata, MD, PhD, MBA.

## Supplementary Tables

**Supplementary Table 1. Results of study quality assessment.**

| Source | Publication date | Study design | Question | | | | | | | | | | % yes | Quality |
|---|---|---|---|---|---|---|---|---|---|---|---|---|---|---|
| | | | 1 | 2 | 3 | 4 | 5 | 6 | 7 | 8 | 9 | 10 | | |
| 1. Ilhan et al. [24] | Feb. 2014 | Case-control study | Y | Y | Y | Y | Y | Y | Y | Y | NA | Y | 87.5% | High |
| 2. Hazra, et al. [25] | Jun. 2022 | Cross-sectional study | Y | Y | N | Y | Y | Y | Y | Y | - | - | 100% | High |
| 3. Albietz, [39] | Nov. 2003 | Retrospective case control study | Y | Y | Y | Y | Y | Y | Y | Y | - | - | 100% | High |
| 4. Albietz et al. [40] | Mar. 2004 | Retrospective case control study | Y | Y | N | Y | Y | Y | Y | Y | - | - | 87.5% | High |
| 5. Albietz et al. [41] | Mar. 2005 | Retrospective case control study | Y | Y | Y | Y | Y | Y | Y | Y | - | - | 100% | High |
| 6. Farahi et al. [42] | Mar. 2014 | Prospective cross-sectional study | **Y** | **Y** | **Y** | **Y** | **Y** | **Y** | **Y** | **Y** | - | - | 100% | High |

| | | | | | | | | | | | | | | |
|---|---|---|---|---|---|---|---|---|---|---|---|---|---|---|
| 7. Wang et al. [44] | Jan. 2016 | Prospective cross-sectional study | Y | Y | Y | Y | Y | Y | Y | Y | - | - | 62.5% | Moderate |
| 8. Maychuk. [43] | Mar. 2016 | Prospective noninterventional cross-sectional study | **Y** | **Y** | **Y** | **Y** | **Y** | **Y** | **Y** | **Y** | - | - | 100% | High |
| 9. Li et al. [46] | Jun. 2021 | Prospective noninterventional cross-sectional study | **Y** | **Y** | N | **Y** | **Y** | N | **Y** | **Y** | - | - | 100% | High |
| 10. Alanazi et al. [45] | Jan. 2021 | Case control study | Y | Y | Y | Y | N | Y | Y | Y | NA | Y | 100% | High |
| 11. Zhao et al. [47] | Jul. 2021 | Prospective cross-sectional study | Y | Y | Y | Y | Y | Y | Y | Y | - | - | 75.0% | High |
| 12. Fagehi et al. [48] | Feb. 2022 | Case-control study | Y | Y | Y | Y | N | Y | Y | Y | NA | Y | 100% | High |
| 13. Fahmy et al. [49] | May 2018 | Prospective cross-sectional study | Y | Y | Y | Y | N | N | Y | Y | - | - | 87.5% | High |
| 14.Yotsukura et al. [50] | Nov. 2019 | Cross-sectional study | Y | Y | Y | Y | Y | Y | Y | Y | - | - | 75.0% | High |
| 15. Upaphong et al. [51] | Jan. 2022 | Cross-sectional study | Y | Y | Y | Y | Y | Y | Y | Y | - | - | 87.5% | High |

Y, yes; N, no; U, unclear; NA, not applicable
